# Primary angiosarcoma of the breast: a case report

**DOI:** 10.1186/1746-1596-8-66

**Published:** 2013-04-22

**Authors:** Amal Bennani, Layla Chbani, Meryem Lamchahab, Mouhcine Wahbi, Fatimazzahra Fdili Alaoui, Ikram Badioui, Moulay Abdelilah Melhouf, Affaf Amarti

**Affiliations:** 1 Department of Pathology, HASSAN II University Hospital, Fez 30000, Morocco; 2 Department of Gynecology and Obstetrics, HASSAN II University Hospital, Fez 30000, Morocco

## Abstract

Primary angiosarcoma of the breast is extremely rare. Radiologic findings are often non specific and may appear completely normal in one-third of cases with primary angiosarcoma. The prognosis is usually poor because of the high rates of local recurrence and early development of metastasis. Surgical removal followed by adjuvant chemotherapy seems improve the prognosis.

We report a case of a 33- year-old woman with a highly vascular mass in her right breast which is suggestive of malignancy at radiology. Initial core needle biopsy showed a benign hemangioma. The patient underwent a mastectomy. The tumor histology showed papillary formations and vascular structures lined by atypical cells with hyperchromatic nucleus and eosinophilic cytoplasm with solid areas. The tumor cells expressed CD34 and CD31 but were negative for cytokeratin. The diagnosis of angiosarcoma grade III was made. The patient is now receiving chemotherapy. She is still alive.

**Virtual Slides:**

The virtual slide(s) for this article can be found here: http://www.diagnosticpathology.diagnomx.eu/vs/1530481200889780

## Introduction

Angiosarcoma of the breast is an exceedingly rare disease that may occur as a primary neoplasm or as a complication of radiation therapy after breast conservation. Only about 20% of angiosarcomas are primary sarcomas. The incidence of primary breast angiosarcoma is about 17 new cases per million women [1].

We report this new case in the aim of avoiding the common trap of a benign differential diagnosis, and achieving a better definition of the treatment of this cancer.

## Case report

We present a case of a 33-year-old woman, with a painful slowly growing mass in her right breast over a period of one year. She had no personal or family history of breast or ovarian cancer. Except the suspect mass, she was in good health.

The physical exam showed an important asymmetry at the expense of the right breast lower area and it was a blackish skin lesion measuring 2 cm in the lower-medial quadrant. The mass was firm and appears to be fixed to the skin. It measures 13 × 12 cm. No axillary lymphadenopathy was palpated. An ultrasound showed a diffuse and ill delimited hyperechogenic infiltration in the inferior portion of the right breast which is hypervascular on doppler sonography. Mammography showed a non specific and diffuse density area of about 12 cm. There was no microcalcification or distortion. The conclusion of radiologist was malign finding (BI-RADS4-5). A core needle biopsy (CNB) was performed and showed non atypical vascular lesion; this was interpreted as a benign capillary hemangioma. This discrepancy between radiological finding and histological results led to the necessity of a macro biopsy.

However, as the mass was so large and highly vascular at Doppler sonography, macrobiopsy was difficult to perform. As consequence, radical mastectomy became more appropriate.

**Figure 1:**
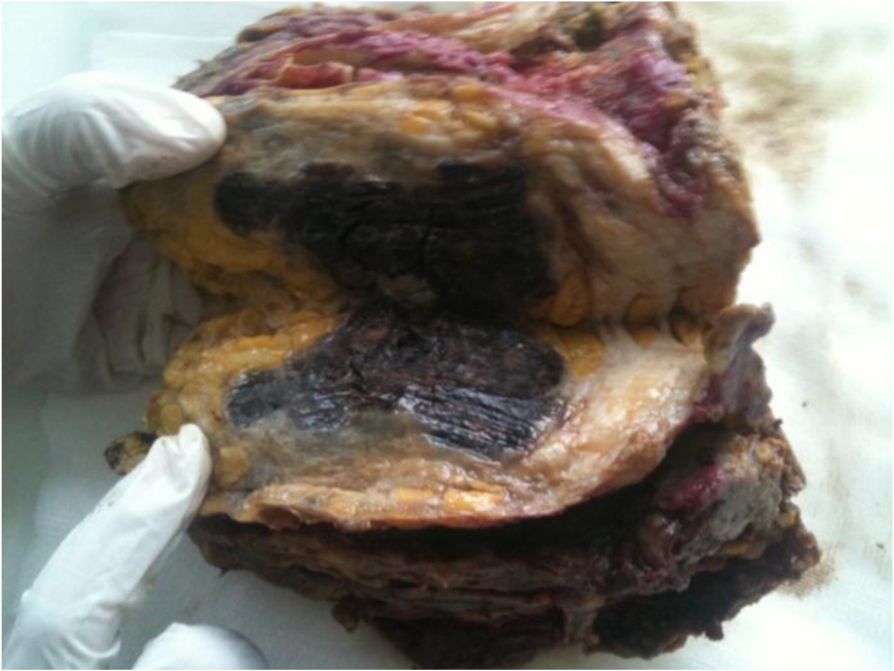
A blackish hemorrhagic ill defined mass of lower quadrants.

At gross examination, the mastectomy measured 16 × 12 cm. The tumor entirely replaces the lower quadrants. It was blackish, hemorrhagic and measures 9,2 cm in the greatest dimension (Figure 1). There were mastopathy lesions with cystic in the upper quadrant.

**Figure 2:**
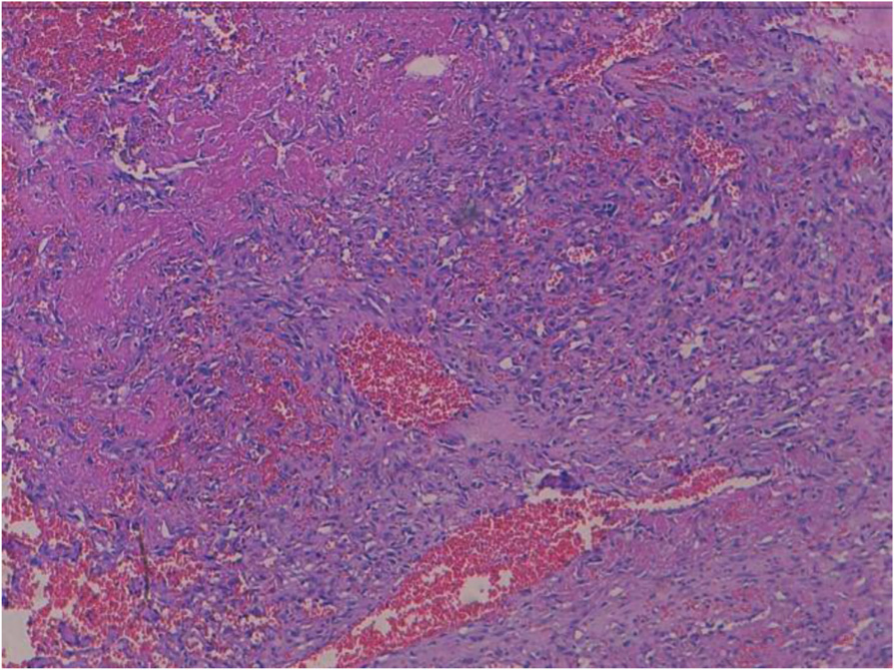
The tumor was made bay papillary formations and vascular structures (hematoxylin-eosin-safran x 5).

**Figure 3:**
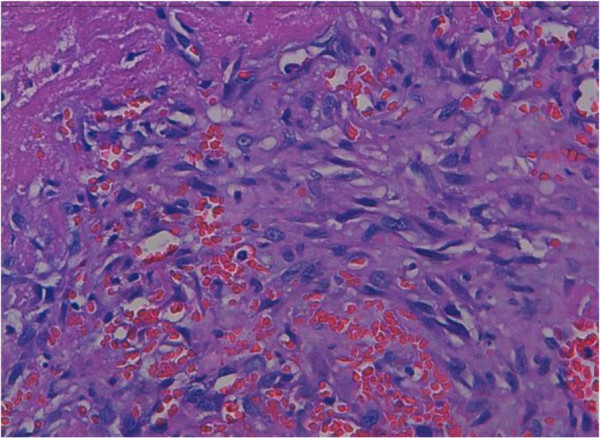
Vascular structures was lined by atypical cells with hyperchromatic nucleus and cytoplasm (hematoxylin-eosin-safran x 40).

**Figure 4:**
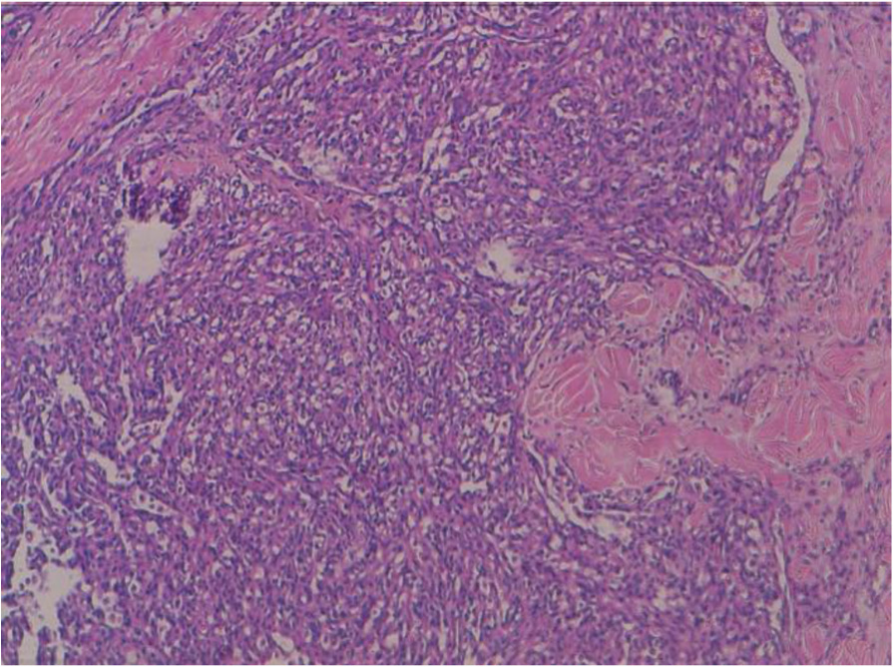
There were solid areas made of spindle cell (hematoxylin-eosin-safran x 10).

**Figure 5:**
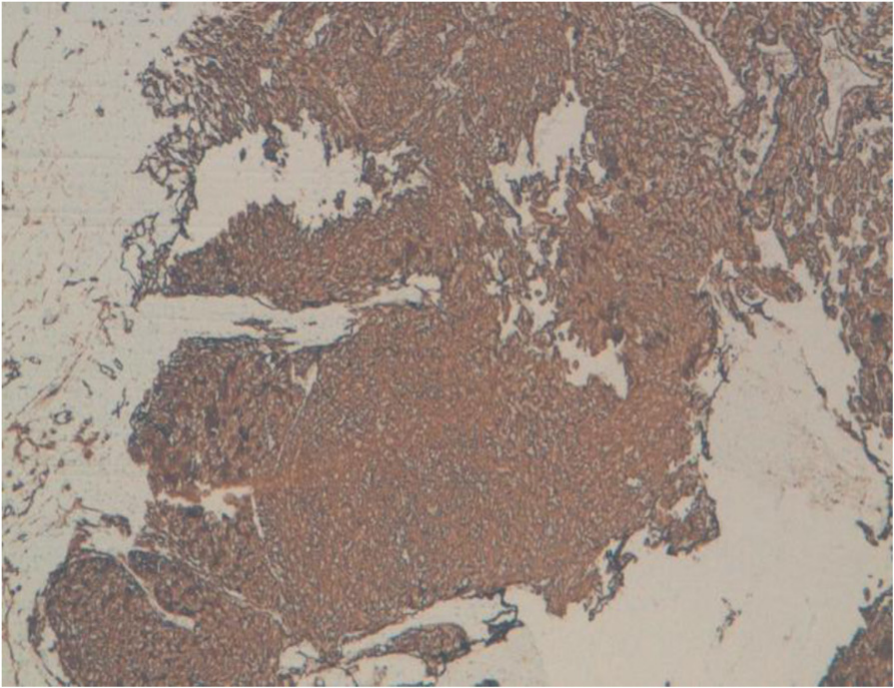
Immunohistochemical stains showed diffuse positivity for CD34 in the neoplastic cells.

**Figure 6:**
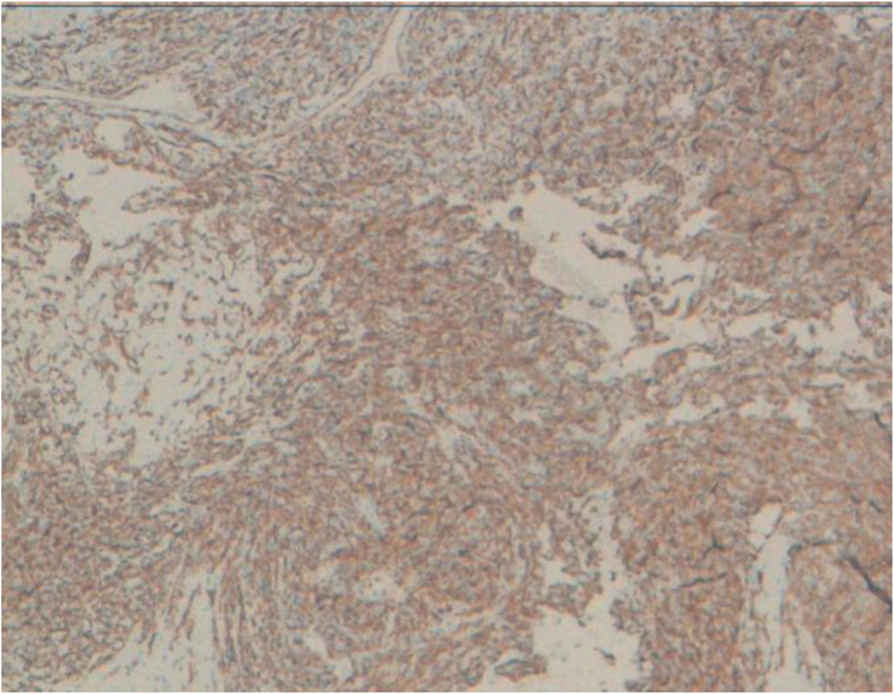
Immunohistochemical stains showed diffuse positivity for CD31.

**Figure 7:**
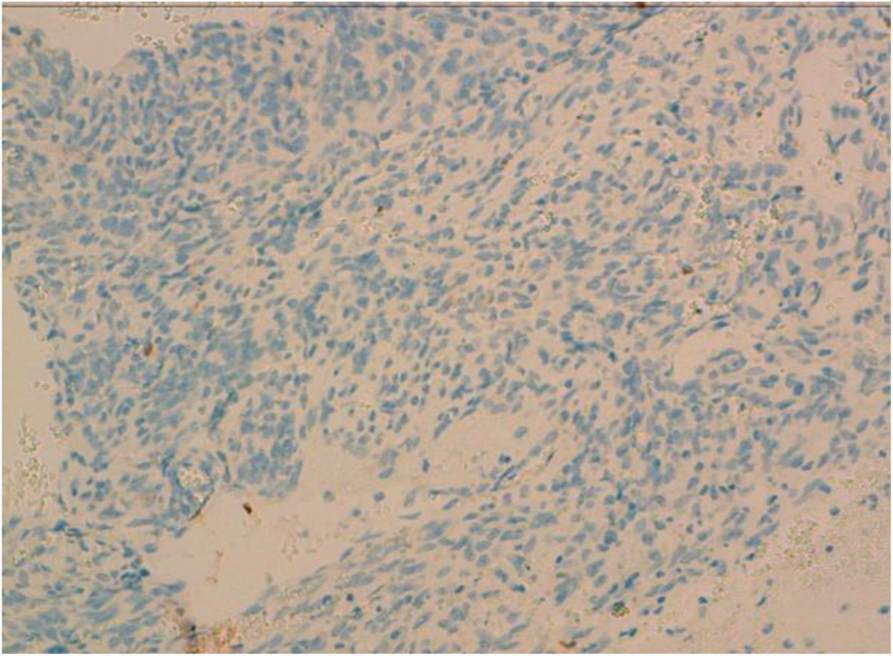
Immunohistochemical stains showed negativity for CK in solid areas.

Histologically, the tumor was made by papillary formations and vascular structures lined by atypical cells with hyperchromatic nucleus and eosinophilic cytoplasm. Mitoses are estimated to 13mitoses/10 high-power magnification (Figures 2 and 3). There were solid areas made of spindle cell mostly devoid of vascular formations (Figure 4). Areas of hemorrhage, known as “blood lakes” and necrosis are also seen. The tumor invades the skin and causes its ulceration. Tumor cells are stained with CD31 and CD34 (Figures 5 and 6). Cytokeratin AE1/AE3 was negative in solid areas (Figure 7). The diagnosis of grade III angiosarcoma of the breast was made. The surgical margins were free of tumor.

Total body CT scan didn’t show any metastasis. Adjuvant chemotherapy was prescribed to our patient. She is still alive.

## Discussion

Mammary sarcomas are a heterogeneous group of malignant neoplasms that arise from the mammary stroma [2]. Angiosarcomas, one of the most common forms of mammary sarcoma, are developed from the endothelial lining of the blood vessels [3].

Breast angiosarcoma can be observed as a primary neoplasm or, more commonly, is described in upper limb lymphedema as a result of mastectomy and radiotherapy for breast carcinoma [4]. Both primary and secondary breast angiosarcomas carry a prognosis worse than mammary carcinoma [5]. Synchronous bilateral angiosarcoma has been reported [5]. In the present paper, in accordance to the case reported, only primary angiosarcomas will be discussed.

Primary angiosarcoma of the breast is exceedingly rare, and represents around 0.04% of malignant breast neoplasms. Its incidence among breast sarcomas varies from 2.7% to 9.1% [6]. Breast angiosarcoma is more frequent in young women (20 to 50 years) like in our case with no previous cancer history or other known risk factors [7,8].

Between 6 and 12% of primary breast angiosarcomas are diagnosed during pregnancy or shortly after, suggesting hormones involvement. However, cases reported to display positive estrogen receptors are so rare that it is presently impossible to establish a link between angiosarcomas and hormonal dependency [9,10]. In the case reported here, estrogen and progesterone receptors were both negatives. Therefore, the role of estrogen dependency in angiosarcoma has yet to be proven and could be subject to future research.

The right breast is more commonly involved than the left breast [11].

In most published cases, breast angiosarcoma is presented as a palpable mass, without pain and with a fast growing rate [11,12].

Radiographically, breast angiosarcomas exhibit no pathognomonic features. They often appear as ill-defined masses on mammograms. Calcifications can be seen but differ from those seen with breast carcinomas [11]. In a review of radiologic findings with angiosarcomas, Liebermane and et al established that the echotexture of these lesions is highly variable. They conclude that Patients with higher-grade lesions at pathologic evaluation were significantly (P less than .05) more likely to have abnormal mammograms, like in our case [13].

In most cases, tumor size at diagnosis is larger than 4 cm [9]. Angiosarcomas larger than 5 cm are associated to a shorter disease-free survival than angiosarcomas smaller than 5 cm. Indeed, tumors smaller than 5 cm are usually associated to a better prognosis, even in the presence of worsening factors.

Diagnosis prior to surgery, either by FNA (Fine needle aspiration) or NCB, is always difficult [9]. Chen and et al. reported a percutaneous biopsy false-negative rate of 37% [6]. The final diagnosis in doubtful cases was made by excisionnal biopsy or was based on the patient's clinical course, which is characterized by episodes of tumor recurrence [6,14].

In our case, Biopsy showed a benign hemangioma but at radiology the tumor was ill defined and seemed more aggressive than a simple hemangioma. So Large-core macrobiopsy was mandatory. But as the tumor was highly vascular at doppler sonography, even macrobiopsy was very difficult to perform, consequently the mastectomy was done.

After mastectomy, further review of biopsy showed a few and focal vascular structures lined by atypical cells without papillary or spindle areas. Those features were not sufficient to confirm the malignancy.

Pathologically, these tumors are subdivided into three groups according to the classification proposed by Donnel and et al [15]. Grade I (well differentiated) contains open anastomosing vascular channels that proliferate within dermis, subcutaneous tissue or breast tissue. A single layer of endothelial cells lines these channels, which dissect through the stroma, causing distortion but little destruction of the preexisting lobules and ducts. The endothelial cells are usually flat; the nuclei may be hyperchromatic and contains small nucleoli. Solid and spindle cell foci, blood lakes, and necrosis are not present. Intermediate-grade angiosarcoma differs from low-grade by containing additional cellular foci of papillary formations and/or solid and spindle cell proliferation. The greater part of the tumor, however, is still composed of low-grade histology. Slightly increased mitotic activity is observed. In Rosen's study, intermediate-grade angiosarcomas behave more like low-grade sarcomas [16]. In grade III endothelial tufting and papillary formations are prominent. Conspicuous solid and spindle cell areas, mostly devoid of vascular formations, are present as well. Mitoses may be brisk, especially in more cellular areas. Areas of hemorrhage, known as “blood lakes,” and necrosis are also seen.

High grade angiosarcoma may contain low or intermediate grade elements, especially at the periphery of the tumor. These elements have deceptively benign appearing and are well differentiated [7]. This explains the fact that the majority of NCB biopsies are negative.

The endothelial cells show reactivity for several markers, including CD31, CD34 and von Willebrand factor (factor VIII). Among them, CD31 is considered the most sensitive and most specific endothelial cell marker.

Some papers have documented that epithelioid angiosarcoma can express cytokeratin and P63 [17].

Differential diagnosis of this rare tumor include: benign hemangioma, phyllodes sarcoma, stromal sarcoma, metaplastic carcinoma, fibrosarcoma, liposarcoma, squamous cell carcinoma with sarcomatoid features, myoepithelioma, fibromatosis, reactive spindle cell proliferative lesion [9], and high-grade mammary carcinoma especially in small biopsy specimens containing only solid areas. Immunohistochemical stains for epithelial markers (pancytokeratin), endothelial markers (CD34 and CD31), and other sarcoma markers should help in making the correct diagnosis.

Surgery is the principal mode of treatment for primary angiosarcoma of the breast and generally consists of a total mastectomy. Hematogenous dissemination is the rule, making axillary lymph node dissection unnecessary. Chemotherapy is observed to be beneficial in high-grade lesions and in the metastatic setting. Preoperative radiotherapy is not indicated in the treatment of angiosarcoma [11].

The degree of differentiation has a significant prognostic value, with regard to both local failure and metastases. Well-differentiated tumors (grade I) have a better prognosis and a higher survival with lower metastatic rate. The prognosis for cases of moderate differentiation is not clear due to the limited number of cases. Other characteristics of the tumor which are of lower prognostic value include: cellular appearance, infiltration of the border, number of mitoses and stromal atypia.

## Conclusion

Young women with solid-appearing breast tumors that are highly vascular at the time of biopsy should be considered malignant until proven otherwise. Donnel's method for grading breast angiosarcoma is easy to implement and correlates well with clinical outcome.

## Consent

Written informed consent was obtained from patient's parents for publication of this case report and any accompanying images.

## Competing interests

The authors declare that they have no competing interests.

## Authors’ contributions

AB, LC, ML, IB and AA performed the histological examination of the breast and were major contributors to writing the manuscript. MW, FF and MAM are the surgeons who operated on the patient and interpreted the patient data. All authors read and approved the final manuscript.
